# Manifestation of glucose-6-phosphate dehydrogenase deficiency in the wake of new-onset type 1 diabetes mellitus: a case report

**DOI:** 10.1186/s13256-022-03549-7

**Published:** 2022-08-26

**Authors:** Sandhya Govindarajan, Imran Zamir, Sunil Bagewadi, Emily Moore

**Affiliations:** 1 Department of Paediatrics, North Manchester General Hospital, Manchester, England; 2 North Manchester General Hospital, Manchester Foundation Trust, Manchester, England; 3 Northern Care Alliance, Manchester, England; 4 Paediatrics and Neonates Trainee, North Manchester General Hospital, Manchester, England

**Keywords:** Diabetes mellitus, G6PD deficiency

## Abstract

**Background:**

Diabetes mellitus is the most common metabolic disease globally, while glucose-6-phosphate dehydrogenase deficiency, an X-linked inherited disorder, is the most common erythrocyte enzyme defect. The association between the two in children has been infrequently reported.

**Case presentation:**

We report the case of a 10-year-old boy of Iraqi descent who presented to our emergency department with new-onset type 1 diabetes mellitus without Diabetic Keto Acidosis. He was treated with subcutaneous insulin and discharged. Eleven days after hospitalization, he was found to be jaundiced during his home visit. Hence, he was referred to the pediatric unit, and his hemoglobin had declined from 130 g/L at the previous admission to 81 g/L. Blood tests revealed low haptoglobin, and his peripheral blood film showed anisocytosis, polychromasia, and occasional red cell fragments suggestive of acute hemolysis. His glucose-6-phosphate dehydrogenase activity was very low, and his subsequent genetic tests confirmed Mediterranean-type glucose-6-phosphate dehydrogenase deficiency.

**Conclusion:**

Glucose-6-phosphate dehydrogenase deficiency in people with diabetes mellitus has been underreported in the literature so far, and screening of glucose-6-phosphate dehydrogenase deficiency should be considered on diagnosis of diabetes mellitus, especially in boys of African, Mediterranean, or Asian descent.

## Background

Type 1 diabetes mellitus, a common metabolic disorder, has a global prevalence of 5.9 per 10,000, with a prevalence of 9.6 per 10,000 in Asia and 5.3 per 10,000 in Africa [1]. The association between diabetes mellitus and hemolysis due to G6PD deficiency has been reported previously, but the underlying mechanisms are not yet fully understood. Glucose-6-phosphate dehydrogenase (G6PD) is an enzyme that makes up part of the pentose phosphate pathway. It maintains adequate levels of nicotinamide dinucleotide phosphate (NADPH) in red blood cells. NADPH plays a role in ensuring there is adequate glutathione to allow the cell to withstand oxidative damage and therefore prevent hemolysis. A reduction or deficiency of G6PD enzyme activity leads to an increased risk of hemolysis, particularly in the presence of oxidative stress. The condition has X-linked inheritance. Heterozygous females are usually unaffected, and there are varying degrees of severity depending on the levels of enzyme activity [2]. Worldwide, 400 million people are affected, most commonly from Africa, Middle East, Asia, Papua New Guinea, and the Mediterranean [2]. Most people with this condition are asymptomatic until hemolysis is induced by known triggers such as fava bean ingestion (broad beans), certain drugs, sepsis, renal failure, and Diabetic Keto Acidosis (DKA) [2].

## Case presentation

We describe the case of a 10-year-old Iraqi descent boy who presented to our emergency department with a history of lethargy, weight loss, polydipsia, and polyuria for 2 weeks. He was born in the UK to parents of Iraqi origin. His past medical history included nocturnal enuresis and an adenotonsillectomy. He is one of five children, and there is no family history of any medical condition. He was not on any regular medications. His weight at presentation was 56.6 kg. His initial laboratory blood glucose was found to be raised (42.9 mmol/l), and venous blood gas revealed a pH of 7.380 (7.35–7.45), bicarbonate 21.3 (21–28) mmol/L, and base deficit of 3.3 (2 to +3) mmol/L. His urine ketones were found to be negative. He was diagnosed with new-onset type 1 diabetes mellitus (DM) presenting without DKA. His blood results showed hemoglobin of 130 (111–147) g/L, white blood cell count of 8.1 × 10^9^/L (4.5 × 10^9^/L to 14.5 × 10^9^/L), and platelet count of 274 × 10^9^/L (150 × 10^9^/L to 450 × 10^9^/L). His serum sodium was 127 (136–145) mmol/L. His renal function tests, liver function tests, and thyroid profile were normal. He was found to have positive islet cell antibodies, and his glutamic acid decarboxylase antibodies were higher than 2000 (<5) U/ml. His celiac screen was found to be negative, and HbA1C level was elevated [116 mmol/mol (20–41 mmol/mol International Federation of Clinical Chemistry standardization)].

He was admitted to hospital, where he underwent a regime of subcutaneous insulin injections and health education around the condition. He was discharged on basal bolus regimen of insulin injections aspart (NovoRapid) and glargine (Lantus) after 5 days.

Following diagnosis, 11 days later, on a home visit by the Paediatric Diabetic Specialist Nursing (PDSN) team, his blood sugar level was found to have returned to normal. During the visit, he was found to be jaundiced with icteric sclera. He also reported extreme lethargy and malaise. He was referred to the acute pediatric observation and assessment unit. On examination, there was no hepatomegaly, and other than icteric sclera there were no stigmata of chronic liver disease. Clinical observations were all within normal limits. Blood results obtained shortly after arrival showed hemoglobin of 81 (111–147) g/L with a low red blood cell count 2.65 × 10^12^/L (3.9 × 10^12^/L to 5.2 × 10^12^/L). His urea and electrolytes were normal. Liver functions revealed raised bilirubin—88 (3–21) μmol/L with a conjugated fraction of 6 μmol/L. His serum alkaline phosphatase, alanine transaminase, C-reactive protein, and serum albumin levels were within the normal range.

## Further investigations

The combination of the sudden drop in hemoglobin from 130–81 g/L within 11 days with jaundice prompted further investigation with regard to cause of his anemia. His blood film revealed anisocytosis, spherocytes, polychromasia, and occasional red cell fragments suggestive of acute hemolysis. Further blood workup showed lactate dehydrogenase levels of 243 (125–243) units/L and low haptoglobin levels of < 0.08 (0.50–2.00) g/L.

## Differential diagnosis

He was investigated for causes of hemolytic anemia and jaundice such as hereditary spherocytosis, thalassemia, autoimmune hemolytic anemia, viral hepatitis, and G6PD deficiency. His serum iron levels, transferrin levels, and coagulation profile were within normal limits, and his hemoglobinopathy screen showed no evidence of thalassemia or abnormal hemoglobin. Direct agglutinin test was negative. His G6PD assay by spectrophotometry showed significantly low activity, that is, < 0.4 (4.8–13.6) IU/gHb. Virology testing showed a negative test for human immunodeficiency virus (HIV), hepatitis A, hepatitis B, and hepatitis C. There was evidence of past infection with Epstein–Barr virus (EBV), cytomegalovirus (CMV), and parvovirus but not of acute infection. Diagnosis of G6PD deficiency was confirmed following the enzyme assay result, which was available 2 days later. The patient remained in hospital until this diagnosis was made and remained clinically stable. His genetic results later confirmed hemizygous G6PD c.653C>T; p. (ser218phe) pathogenic variant, consistent with the diagnosis of Mediterranean-type G6PD deficiency.

## Treatment

He did not undergo blood transfusion but was commenced on folic acid in view of his active hemolysis by the hematology team. The rationale behind prophylactic administration of folic acid is that acute hemolysis can consume folate and lead to megaloblastic anemia [3]. During his stay, repeat full blood counts were performed that showed a rising trend in hemoglobin. Four days following admission, hemoglobin had risen to 89 g/L and reticulocytes were at 22.8%. He was discharged on folic acid 5 mg once a day for 12 weeks with planned follow-up with diabetes team and hematology team.

## Outcome and follow-up

Blood testing 1 month later showed complete recovery of hemoglobin levels (136 g/L) and liver function tests (serum bilirubin 12 μmol/L).

He was kept under follow-up for diabetes mellitus and G6PD deficiency. Assessment of HbA1C levels repeated after 2 months revealed 45 (20–41) mmol/mol. He is currently on long-acting basal insulin (Degludec) and insulin aspart (Fiasp) for his diabetes and is under follow-up with the diabetic team and hematology team. He successively developed three further episodes of mild jaundice following hemolysis during acute illnesses, including one episode of cellulitis and two episodes of respiratory tract infection, in a period of 1 year, suggesting that G6PD Mediterranean type presents with enzyme deficiencies that are more severe than in the other G6PD variants.

## Discussion

G6PD is involved in the pentose phosphate pathway , which catalyzes the reaction that produces NADPH, by which erythrocytes protect themselves from hemolysis caused by oxidative stress [4]. It also replenishes the reduced form of glutathione, which maintains hemoglobin in reduced form [4]. Hemolysis in patients with G6PD deficiency during treatment of type 1 diabetes mellitus has been reported previously; however, the mechanism behind it is not yet fully understood [5, 6].

To date, 25 cases have been reported from 12 different countries in both type 1 and type 2 diabetes mellitus in patients aged 4 years [6] to 58 years [7]. Hypoglycemia [8], blood glucose normalization [9], ketoacidosis [5], and administration of metformin [10] or glibenclamide [7] are considered as possible causes of hemolysis in patients with DM and underlying G6PD deficiency. Blood glucose normalization after treatment with insulin could have been a likely cause of hemolysis in our patient. During administration of insulin, the drop in blood glucose levels causes NADPH loss in red blood cells, which makes them sensitive to oxidative stress [11, 12]. This, combined with loss of sulfhydryl group availability due to G6PD deficiency, is likely to trigger hemolytic anemia in patients with type 1 diabetes mellitus [12].

Gradual plasma glucose correction to avoid rapid decrease in glucose availability for red blood cells is likely to reduce the occurrence of hemolysis in G6PD-deficient patients [13]. Also, screening of G6PD enzyme activity should be considered in patients newly diagnosed with diabetes, especially in boys, considering the ethnic origin of the patient, to reduce the risk of hemolysis. G6PD deficiency screening is a cost-effective test at a rate of about 3 USD per screening test and is considered economical compared with the average cost of hospitalization due to hemolysis secondary to G6PD deficiency [14].

Glycosylated hemoglobin (HbA1c) is an important determinant of diabetes control and reflects the mean glycemic level over the preceding 120 days, which is the average lifespan of a red blood cell. The HbA1c level at any point in time is contributed to by all the circulating erythrocytes, from the oldest (120 days old) to the youngest [15]. Falsely low HbA1c has been reported in glucose-6-phosphate dehydrogenase deficiency due to increased red cell turn over [15] and reduced exposure time of hemoglobin to glucose [16]. Hence, HbA1C levels may not be a reliable indicator of adequate glycemic control in a patient with type 1 diabetes mellitus with underlying G6PD deficiency.

## Conclusion

1. We hypothesize that, on the basis of the global prevalence of both conditions, G6PD deficiency in people with diabetes mellitus has been underreported in the literature so far, and screening of G6PD deficiency, being a cost-effective test, should be considered on diagnosis of diabetes mellitus, especially in boys of African, Mediterranean, or Asian descent.
2. HbA1C levels might not be always reliable for monitoring glycemic control in a diabetic patient with G6PD deficiency.
3. Hemolysis may occur during normalization of blood glucose levels in a G6PD-deficient patient with diabetes such as after treatment of DKA.

## Data Availability

The data that support the findings of this study are available from the corresponding author (SG) upon reasonable request.
